# S9-2 Dutch national falls prevention program- implementation challenges

**DOI:** 10.1093/eurpub/ckad133.044

**Published:** 2023-09-11

**Authors:** Liesbeth Preller

**Affiliations:** Knowledge Centre for Sport & Physical Activity Netherlands

## Abstract

**Purpose:**

In the Netherlands, a very ambitious falls prevention program is being implemented in 2023 and 2024. Falls among older adults lead to a high burden on healthcare and high societal costs. This program is therefore part of the current government coalition agreement. Each year one in seven persons over 65 should be screened on fall risk and should be stimulated to reduce fall risk. The whole program includes specific falls prevention programs (FPPs) as well as Physical activity (PA) programs outside these FPPs to keep fall risk restricted. Large responsibility is given to municipalities for implementation at the local level, and to regional public health services at a higher level.

**Project description:**

At present there is no good organisation of falls prevention. A whole infrastructure has to be set up to be able to carry out the prevention program. This is coordinated by VeiligheidNL.

It involves a considerable number of stakeholders and different working packages on FFPs, implementation, and communication. Starting in 2022, a number of sessions have been held to inform municipalities, regional health services, and professionals. Funding frameworks are set up including refunding of FPP participation and support of coordination by municipalities.

As part of the process, we evaluated among scientists, PA professionals and municipalities what is needed to organise PA for maintenance after FPPs and for people without increased risk. This includes more available organised PA, increase in frequency and intensity of current group PA, and involving regular sports clubs to increase overall capacity.

Most municipalities need to take a much stronger coordinating role, including also a professional with ‘broker’ role, generally the so-called neighbourhood sports coach (NSC). This professional guides older people to suitable PA and is a connector between health, social care and PA professionals.

**Conclusions:**

The ambitious falls prevention program, supported by considerable financial resources, has the potential to give a large boost to PA in older adults. There are still a lot of challenges at the national as well as local level before implementation is successful, but several locations have already managed to develop a good infrastructure.

